# How decision for seeking maternal care is made - a qualitative study in two rural medical districts of Burkina Faso

**DOI:** 10.1186/1742-4755-10-8

**Published:** 2013-02-07

**Authors:** Donmozoun Télesphore Somé, Issiaka Sombié, Nicolas Meda

**Affiliations:** 1 Société d’Etudes et de Recherche en Santé Publique (SERSAP), Ouagadougou, Burkina Faso; 2 West African Health Organization (WAHO), Bobo-Dioulasso, Burkina Faso; 3 Centre Muraz, Bobo-Dioulasso, Burkina Faso

**Keywords:** Decision-making process, Maternal care, Rural medical district, Burkina Faso

## Abstract

**Background:**

Delay in decision-making to use skilled care during pregnancy and childbirth is an important factor for maternal death in many developing countries. This paper examines how decisions for maternal care are made in two rural communities in Burkina Faso.

**Methods:**

Focus group discussions (FGDs) and individual interviews (IDIs)) were used to collect information with 30 women in Ouargaye and Diapaga medical districts. All interviews were tape recorded and analyzed using QSR Nvivo 2.0**.**

**Results:**

Decision-making for use of obstetric care in the family follows the logic of the family’s management. Husbands, brothers-in-law and parents-in-law make the decision about whether to use a health facility for antenatal care or for delivery. In general, decision-makers are those who can pay, including the woman herself. Payment of care is the responsibility of men, according to women interviewed, because of their social role and status.

**Conclusions:**

To increase use of health facilities in Ouargaye and Diapaga, the empowerment of women could be helpful as well as exemption of fees or cost sharing for care.

## Background

Maternal mortality remains high in many developing countries; in Burkina Faso, it is approximately 484 women out 100,000 live births [1]. Certain avoidable factors (biomedical, reproductive, health service factors, socioeconomic and cultural factors) increase the risks of severe complications or maternal death. All these factors contribute to the three delays model [2]. This model attributes the death of many pregnant women in developing countries to three primary factors: the delay in making the decision to seek care when there is a complication; the delay in reaching a health centre once the decision to seek care has been made; and the delay in receiving adequate and appropriate care once a medical facility has been reached.

Some studies of developing nations, especially those in West Africa [3], emphasize the necessity for women to seek spousal or a family member’s permission before be able to seek obstetric care. In some parts of Nigeria, a study [4] has described that spousal permission is important before a woman in emergency obstetric conditions attends to health care. In the absence of the chief of the household, any male must accompany the woman to the clinic; but it seems that women wait for the husband. In the case of Senegal, Dia et al. [5] claim in their study that around 50% of their respondents recognized that decision to seek obstetric care is made by the husband. Women cannot and do not decide on their own to seek care [5]; the decision belongs to the spouse or senior family members. It is accepted that delaying the decision to seek care could be a factor of maternal complications or death [5]. To prevent these complications and deaths, it is important to understand how the decision to seek care during pregnancy or childbirth occurs. The description of the process of decision-making is also important to inform programmes to prevent the delay in care-seeking. For these reasons, it is vital for us to determine what, specifically, goes into the decision-making process when a woman uses a health facility.

In Senegal, Dia et al. [5] have shown in their study that 52% of respondents said that husbands would make the decision and 44% said another member of the family.

The other important factor is financial. They also described that when the couple could not provide money for hospital care, the decision about where to seek care is made by the community leaders who can in such cases override the husbands’ wishes. Traditionally, payment for obstetric services is the responsibility of the husband, independent of the wealth of the woman [5].

Some studies have explored the problem of decision-making in others domains of health such as reproductive health [6,7], nutrition [8] child health [9]; but not specifically in the decision-making process in the family for the use of health services for obstetric care.

The results presented in this paper are a part of an evaluation of a Skilled Care Initiative (SCI), an intervention implemented in 2002 by Family Care International (FCI) in the medical district of Ouargaye. Diapaga was the control district. The intervention aimed to reduce maternal mortality by promoting the use of skilled care at delivery. The evaluation was carried out by Immpact (Initiative for Maternal Mortality Programme Assessment) in 2006.

The study was conducted with the idea that our understanding of factors affecting decision-making processes could improve the use of health centres for obstetric care.

## Methods

Clearance to carry out this study was obtained from the Ethical Committee of Centre MURAZ (CM). Before in-depth interviews (IDI) and focus group discussions (FGD), written individual consent to participate was sought after the study’s aim, methods and benefits were explained to potential participants. The researchers also explained that declining to participate would have no negative implications for care anywhere and that anonymity would be maintained.

The study was conducted from April to May 2006 in Ouargaye and Diapaga medical districts. Five health centres were chosen: Diapaga, Ouargaye, Namounou, Comin Yanga, and Dourtenga. For the choice of health centres, we considered in Ouargaye district, one site where there is the skilled care initiative (Dourtenga), one site where there is not the skilled care initiative (Comin Yanga) and Ouargaye were there is the district hospital. In Diapaga, we choose Namounou (It was the only site similar to SCI site because of the presence of a confessional health centre with good indicators) and Diapaga where there is the district hospital.

The participants were women aged from 15–49 who either used or did not use a health facility for maternal care and from the ethnic groups of Yana for Ouargaye and Gourmanthé for Diapaga.

Before the start of the main study, a pre-test of the method was carried out in 2005 and a pilot in 2006. An interview guide was elaborated for in-depth interviews and focus group discussions. Eight (8) focus group discussions (three in Diapaga and five in Ouargaye) and 30 individual in-depth interviews (nine in Diapaga and 21 in Ouargaye) were conducted. The difference remarked in IDI and FDG numbers was due to availability and willingness to participate. In Diapaga, the presence and the permission of the husband was always required before the woman accepted discussion.

Participants were selected by snow ball method in each village by the liaison of village midwives, who knew pregnant women, women who delivered at health facility and women who gave birth at home.

All interviews were tape-recorded, transcribed and translated into French. They were then prepared and imported into NVIVO, the qualitative software we used for codification and analysis.

## Results

### Decision-making process for utilization of maternal care

**Figure 1:**
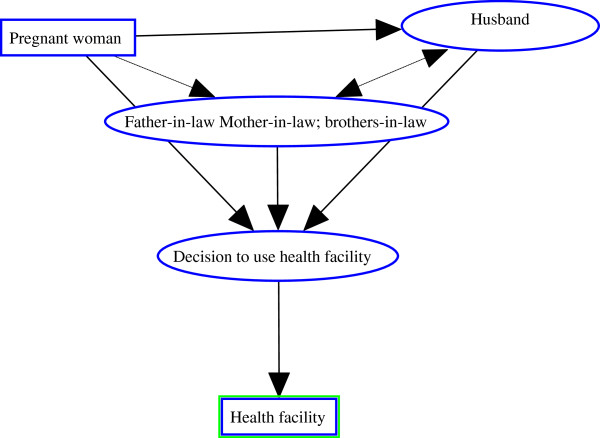
Conceptual framework of decision making process.

The traditional necessity for women to have the permission of husbands or their relatives, before leaving the home, predominates in both districts. This situation pertains to many domains and especially to issues about health. In terms of obstetric healthcare, the use of a health centre is not always possible for pregnant women. To make use of maternal care, they need consent of a member of the family (see Figure 1). A respondent from Comin Yanga in Ouargaye district stated

"“I inform the person to whom I belong; it is my husband that I inform ( ) when I want to go for weighing, I inform him. I inform him also when it is time for the children to be weighed.” DR_ Comin yanga"

Many elements contribute to decision-making surrounding maternal healthcare. The woman can inform her husband that something is wrong and that she needs some care. The husband can decide to let her go to a health centre if he estimates that the woman is really suffering and there are some signs of complications like bleeding. At this stage, the husband can decide and inform his relatives. But he can request the opinion of his relatives, specifically his mother. If she estimates that there is no complication and the case can be managed locally, the use of a health centre is abandoned. The power of the mother-in-law in this context is related to distribution of rules in traditional societies and the fact that elder women play an important role for the cohesion and the management of households by their advice on how to take care of the whole family and specifically young children.

Alternatively, the woman experiencing a health problem can first approach the mother-in-law. When the mother-in-law is convinced, she informs her son that a trip to the health centre is a necessary recourse. This is described in the words of a respondent from Dourtenga (E_Dourtenga):

"“Apart from the head of family, it is our mother-in-law who decides. She can suggest it to his son if she notices something wrong”."

In general, a mother-in-law’s advice is respected due to their own childbirth experience; so she can oblige her son to take the woman to the health centre. When a woman’s decision to go to a health centre is overridden because there is no money, another decision can be made. In both districts, women sometimes sell goods and can have some money of their own. When they inform the husband or his relatives, and there is a delay or a refusal to make the decision because of lack of money, the woman decides herself to go to the health centre. In such cases, she will pay all the costs herself. Z. from Dourtenga (Ouargaye) said:

"“You inform your husband who takes you to a health facility. In some situations you can be ill and he will not pay attention to you. Even if you died he will not be interested in you, except if you yourself decided to take the decision to go to a health facility”."

The situation can be particular for single women or widows. In the two districts, there is some situation where women are responsible of a household but they referred always to some relatives. It is the cases of widows and single women. Even they did not have à formal man, but there is someone who is responsible of the pregnancy and makes decision about this.

### Requirement of permission and care payment responsibility

In both districts as in many others societies, women get married and stay in the husband’s household. In Diapaga district, traditional practice is that a young man can kidnap a young girl and oblige her to be his wife. The family of the young girl is later informed and arrangements are made for the marriage. For such marriages, the consent of the young girl is not necessary. Marriage is most of the time an arrangement between two families and the woman is accorded no legitimate point of view. She accepts the situation. In these conditions, she is supposed to belong to her husband and his family. So, when she wants to do something extraordinary, she needs the permission of the husband or his relatives or a member of the family. R. in Comin Yanga (Ouargaye) expressed the situation in these words:

"“I inform the person to whom I belong”."

For women in both districts, it is a question of respecting the matrimonial link. A. from Namounou (Diapaga) stated

"“Because he takes care of me, I cannot just go; it is a question of respect”"

Women and their children belong to the husband. This situation does not permit women to participate effectively in decision-making concerning their health. The mother-in-law, when she is sensitive to the situation, can be a good partner. She can play an important role in the wellbeing of the woman if their relationship is good. In the household, she can control the conflicts when there are several wives, manage the distribution of food and play the role of regulator of family wellbeing and is generally the elder woman in the household. Otherwise, when the relationship is not good, the woman can be considered as a lazy person and no attention will be paid to her.

Women also request permission before seeking maternal healthcare for financial reasons. In order to gain the support of the husband, it is important to have his agreement before seeking care. In Ouargaye, D. (Dourtenga) described the situation in these words:

"“How can you just go to the CSPS (health centre) without the permission of the head of the family? Because sometimes, he gives you money for weighing; but if you do not tell him, you cannot have money for care….”"

The fact that women cannot decide by themselves to use health centres or to go out of homes without permission from the family-in-law, constrains them into a dependency on men.

Traditionally, men are the managers of all financial matters of the family; so, from the viewpoint of women, they have to pay for all the expenses. As expressed by this woman from Comin_yanga

"“As it is him who takes care of you, he has to pay for the household expenses; he must pay for all the expenses” F_Comin Yanga."

Another view expressed by women is that each partner has his role and theirs is to manage the pregnancy and the childbirth. Payment is perceived as an obligation for the husband and his family according to M. from Comin Yanga

"“Yeah! It is the head of the family! who will pay for care? He is the father of the pregnancy (laughing). As he is the father of the pregnancy, he must pay for the table [table used in the maternity ward during the delivery] and drugs.” M_Comin Yanga."

The same point is expressed by Y. from Namounou

"“… because he takes me to his home, he must now take care of everything and the child is for him. So it is an obligation for him.”"

## Discussion

According to the results from the FGDs and IDIs, there are two main problems: One is poverty, which prohibits the family from seeking obstetric care. The second is a lack of an empowerment of women. From the viewpoint of the women interviewed, the decision-making process to use a health facility for skilled care is mainly managed by the husband, his parents (mother- or father-in-law) or his brothers.

A study carried out in rural Tanzania, resulting in similar findings, has shown that decision makers in maternal referrals are husbands and relatives [10]. The process in deciding to seek referral care is influenced by a community’s perception of the seriousness of the condition, the difficulty of access and the cost involved in transport, living expenses at the hospital, and perceived quality of care at the facility level. In our study, the husband and his relatives were also found to be the main decision-makers, the reason for this being that the husband is the manager of the income of the family. He is the head of the family and all matters concerning the household must have his clearance [11]. This situation is favored by tradition; generally men are considered more powerful than women in society; the consequence is that women do not have enough power to participate in the decision-making concerning the household and their health. They refer to the husband or the mother-in law. The importance of the mother-in-law in this process is that in some situations, several households live as one family with the mother-in-law as a key person for all things concerning women (such as cooking, pregnancy and delivery, health of newborn, children and women). She is perceived as a model for the household wives and also the evaluator of the behavior of the wives. In general, the mother-in-law is much respected and her viewpoint is important in the decision-making process. Generally problems of health, sex and reproduction related to women are managed by the mother-in-law. Obstetric problems, generally called “women’s problems” are not often discussed with partners but with the mother-in-law or mothers. When the mother-in-law perceived the importance and the need to use health care, she can be a facilitator and aid for the choice to use a health facility. If not, the mother-in-law can decide that the woman can give birth without the assistance of the clinic. This happens when the mother-in law herself gave birth at home for all her pregnancies without apparent complications. A daughter-in-law could be considered as a lazy woman if she insisted on delivering in a health facility. Also, as has been shown in the case of the Gambia, post-menopausal women are seen as experts on pregnancy and childbirth and are consulted if a complication is noticed during pregnancy, labor or during the puerperium; their words are hardly challenged in Gambian society [12].

The low status of women has consequences for the decision-making process. Delay in reaching the health facility is sometimes catastrophic for women [13]; but what can they do if they cannot always decide by themselves?

In both districts, in general, men get married to have children and to have someone to help them for agricultural work. This situation sometimes does not take the care for women’s health into account.

Traditions heavily influence the decision-making-process; women play a secondary role to men in this culture. In our study, all the women living in couples have recognized that expenses are the responsibility of the husband. It means that society, traditions do not provide enough power to women to make them autonomous. It has been described in a study [5]that payment of antenatal clinic bills might not even be associated with the wife’s ability to pay, but as a traditional responsibility of the husband. No matter how financially stable a woman is herself, it is the responsibility of the husband to pay the antenatal care bills and all bills associated with the emergency obstetric conditions. The reasons evoked are related to the role of each partner. Women attributed to themselves the role of reproduction. In return, they do not have to suffer to find money for care. But the problem is if men do not have money since the decision-making to go to a health facility is related to money. When there is money, it is possible for man to decide to go to a health facility for care; but, if there is no money, it is difficult for the man to decide to go; if they go, how will he pay for care and drugs? So, we think that some actions must be taken to help women to be somewhat autonomous financially by helping them to implement small projects in agriculture or cattle farming or others things which could be helpful for them in their context. The empowerment of the women could be such an opportunity.

The empowerment of women (both daughters-in-law and mothers-in-law) is vital to the reduction of maternal mortality. Both groups need to be involved in strategies to strengthen their power. It is true that in this context, the most important thing is the internal empowerment of daughters-in-law in opposition to mothers-in-law. It is equally true that, in some situations, mothers-in-law contribute to the poor status of daughters-in-law. But in the process of empowering women, what can be done? The empowerment of women is a process in which many elements need to be considered. But, the most important area in which an impact can be made in our context is to facilitate the right for women to have access to land and to improve their economical status. In both districts, women cannot be landowners even while the economy of the country is based on agriculture. The relationship between the capacity to pay and decision-making could be overridden, if women would have a better status in the society.

It is now clear that to reach the fifth Millennium Development Goal (MDG 5), whose objective is to reduce maternal death by 75% by 2015, urgent strategies have to be implemented not only in the reinforcement of health supplies but also at the demand level by considering the factors that prevent women’s use of health facilities.

## Conclusions

This study has shown that the decision to use obstetric care in rural Burkina Faso is mainly made by men and mothers-in law, therefore, any initiatives to increase the use of clinical obstetric care should involve men, specifically husbands and partners and the woman’s mother-in-law.

The reduction of maternal mortality as mandated by the international community through the MDGs, will not be achieved if steps are not taken to empower women and particularly pregnant women by reducing their poverty [13]. The use of health facilities is one of the conditions necessary for every woman to benefit from skilled care and to minimize the risk of maternal death. To increase the use of health facilities, women and specifically men must have the information regarding the importance of seeking skilled care during pregnancy and childbirth. Community mobilization should be helpful to give information to people particularly men and mothers-in-law, etc. on the advantages of using health facilities. The government must waive the antenatal care, childbirth, C-section and newborns care for all women.

The study showed that men are responsible for payment during pregnancy and delivery but it is not always possible to have money to pay for care. So, having another source of revenue in the family is very important. Women could help with this if they were empowered with the means to make more money. It is important that women participate in the decision-making process for questions related to their life and their health.

This study has some limits related to difficulty to discuss with women in the two districts. Sometimes it was very hard to discuss with only the woman without the presence of the husband or another member of the family. But these difficulties reinforce the conviction of the importance of decision-making power for women.

## Competing interests

The authors declare that there are no competing interests. The evaluation was financed by the Bill and Melinda Gates Foundation, the Department for International Development, the European Commission and USAID.

## Authors' contributions

TDS (SERSAP) – participated in the study design, conducted anthropological fieldwork, conducted analysis of anthropological data, participated in analysis of findings, conceptualized the paper, and wrote the first draft of the paper. IS (WAHO) – participated in the comments of the structure and content of the paper. NM (Centre Muraz) – participated in the comments of the structure and content of the paper. All authors read and approved the final manuscript.
